# Cellular Delivery of Doxorubicin via pH-Controlled Hydrazone Linkage Using Multifunctional Nano Vehicle Based on Poly(β-L-Malic Acid)

**DOI:** 10.3390/ijms130911681

**Published:** 2012-09-17

**Authors:** Rameshwar Patil, Jose Portilla-Arias, Hui Ding, Bindu Konda, Arthur Rekechenetskiy, Satoshi Inoue, Keith L. Black, Eggehard Holler, Julia Y. Ljubimova

**Affiliations:** 1 Nanomedicine Research Center, Department of Neurosurgery, Cedars-Sinai Medical Center, 110 N. George Burns Rd. Davis Building, Room 2094-A, Los Angeles, CA 90048, USA; E-Mails: portillaj@cshs.org (J.P.-A.); dinghx@cshs.org (H.D.); kondab@cshs.org (B.K.); rekechenetskiya@cshs.org (A.R.); inoues@cshs.org (S.I.); blackk@cshs.org (K.L.B.); ljubimovaj@cshs.org (J.Y.L.); 2 Institut für Biophysik und Physikalische Biochemie der Universität Regensburg, Regensburg 93053 Germany; E-Mail: hollere@cshs.org

**Keywords:** polymalic acid, doxorubicin, nanoconjugate, pH-controlled hydrazine linkage, brain and breast cancer

## Abstract

Doxorubicin (DOX) is currently used in cancer chemotherapy to treat many tumors and shows improved delivery, reduced toxicity and higher treatment efficacy when being part of nanoscale delivery systems. However, a major drawback remains its toxicity to healthy tissue and the development of multi-drug resistance during prolonged treatment. This is why in our work we aimed to improve DOX delivery and reduce the toxicity by chemical conjugation with a new nanoplatform based on polymalic acid. For delivery into recipient cancer cells, DOX was conjugated via pH-sensitive hydrazone linkage along with polyethylene glycol (PEG) to a biodegradable, non-toxic and non-immunogenic nanoconjugate platform: poly(β-l-malic acid) (PMLA). DOX-nanoconjugates were found stable under physiological conditions and shown to successfully inhibit *in vitro* cancer cell growth of several invasive breast carcinoma cell lines such as MDA-MB-231 and MDA-MB- 468 and of primary glioma cell lines such as U87MG and U251.

## 1. Introduction

The anticancer drug doxorubicin (DOX) is potent and therapeutically efficient for treatment of a variety of tumors [1,2]. However, it has considerable toxicity, which limits its therapeutic use, preventing treatment at high dosages, and it has an acquired resistance [3–6] excluding repeated treatment at tolerated dosages.

In recent years, significant efforts have concentrated on nanoscale delivery systems of DOX [7–11]. Compared with chemotherapeutic molecules for cancer treatment, “nanodrugs” offer several advantages such as increased solubility, tumor targeting, enhanced accumulation in tumor tissue and tumor cells, decreased systemic toxicity and increased maximum tolerated dosages. Nanodrugs can selectively accumulate in tumor through a passive targeting mechanism known as enhanced permeability and retention (EPR) effect [12–14]. In addition, an efficient tumor accumulation is achieved through active targeting by combining nanodrugs with specific antibodies that bind to receptors overexpressed on vascular tumor endothelium. Another important targeting is binding to surface antigens which are specific for tumor cells [15–17]. The effect of this targeting will be enhanced internalization of nanodrugs and increased treatment efficacy. While improved delivery through targeting is a general property of various delivery devices, polymer-based delivery systems have also proven advantageous to circumvent multidrug resistance [18,19] and to be less immunogenic than protein based e.g., viral vectors when antitumor treatment is repeated, avoiding acute or chronic host immune response [17,20–22].

The application of various nanodrugs such as liposome and polymer micelle systems in cancer treatment improved DOX therapeutic efficacy and reduced acute toxicity of the free drug. One such successful example is “DOXIL^®^” a clinically approved nanodrug for the treatment of refractory ovarian cancer [23]. Among nanoconjugates used to deliver DOX, the polymer platform *N*-(2-hydroxypropyl)methacrylamide (HPMA) showed promising results in animal models [8] and also entered clinical trials [13]. In this nanoconjugate, the attachment of DOX via an acid-cleavable hydrazone linkage has been an effective way to enhance the delivery of DOX [11,24,25], because the hydrazone linkage is cleaved under the mild acidic conditions of late endosomes/lysosomes to yield free DOX molecules. The drug then diffuses into the cytoplasm after spontaneous deprotonation of the galactosamine residue.

**Figure 1:**
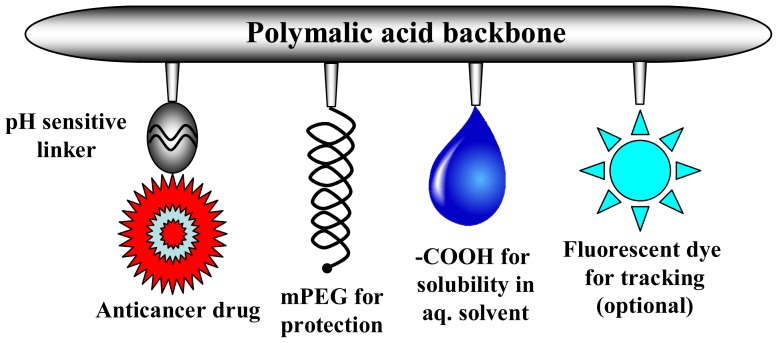
Schematic presentation of the drug delivery system.

We have demonstrated the successful delivery of several anti cancer agents specifically to brain and breast tumor using the targeted nanoconjugate delivery system Polycefin including temozolomide [26] and antisense oligonucleotides [27–29]. This system uses the nanoscale platform poly(β-l-malic acid) (PMLA) known to be biodegradable, non-toxic and non-immunogenic. It provides numerous pendant carboxyl groups accessible for the chemical attachment of drugs and other functional moieties. In order to enlarge our palette for chemotherapeutic drugs, we have chosen here the delivery of DOX. Also, we wished to attach a bactericidal in addition to antisense oligonucleotides and antibodies to be effective during immune compromising anticancer treatments. In this PMLA based delivery vehicle, DOX is bound to the carrier platform via an acid labile hydrazone linker that can be cleaved in the endosomal compartment before delivery to the cytoplasm of the recipient cell. The delivery system is schematically presented in Figure 1. Nanoconjugate contains PEG for protection against resorption by the reticuloendothelial system (RES) and it may optionally contain a fluorescent dye for tracking after systemic injection.

## 2. Results and Discussion

### 2.1. Synthesis of Nanoconjugates

In order to conjugate DOX to a PMLA backbone, the spacer glycine Boc-hydrazide (GBH) was synthesized as illustrated in Scheme I with minor modifications of a method described elsewhere [30]. GBH was obtained by hydrogenolysis of intermediate **3** at atmospheric pressure in the presence of 10% Pd-C in MeOH at room temperature (RT) and recrystallized from ethyl acetate and petroleum ether mixture.

The multi-component drug delivery system was synthesized using PMLA as polymer platform. Synthesis of nanoconjugate proceeded very smoothly and involved two major steps. As presented in Scheme II, the first step resulted in the chemical activation of all the PMLA pendant carboxyl groups with DCC/NHS mixture forming the NHS-ester. Subsequent nucleophilic replacement by mPEG-amine and glycine spacer was achieved with ease forming stable amide bonds. These reagents were used in limiting stoichiometries and their reactions were completed as indicated by TLC/ninhydrin tests. On this basis, the percent loading corresponds with the amounts of reagents. Remaining unused activated ester was then hydrolyzed under acidic pH (5.5); during this process the Boc group of glycine spacer was also removed.

To examine whether the removal of the Boc group was complete, the product was treated with 20% TFA in DMF for 2 h, a reaction known to completely eliminate the Boc group. Sec-HPLC did not show any difference for products before and after treatment with TFA (data not shown). DOX was then conjugated in the second part of synthesis; it was reacted with preconjugate in the presence of activated molecular sieves in the dark forming stable hydrazone bond. Excess amount of DOX was used in this conjugation reaction, and unreacted DOX was then removed by preparative size exclusion chromatography. After purification, freeze-dried intermediates and products were stored at minus 20 °C for several months without any change in physicochemical properties.

### 2.2. Characterization of Nanoconjugates

Hydrodynamic diameter and ζ-potential were measured at 25 °C using the Zetasizer Nano ZS90 System (Malvern Instruments, Malvern, UK), and the results are summarized in Table 1. The hydrodynamic diameter of P/PEG(5%)/GH-DOX(5%) could not be measured, because of the interference of DOX fluorescence with the light scatter acquisition. According to the protocol provided by Malvern, the hydrodynamic diameter that was measured by Dynamic Light Scattering (DLS) refers to a sphere that has the same translational diffusion coefficient as the studied nanoconjugate. Thus, the feature of an elongated structure like the one assumed for the nanopolymers would not be indicated. Another observation with our nanopolymer was the relatively high PDI-value after conjugation with PEG or other molecules. Since we do not find variations in SEC-HPLC elution profiles before and after conjugation that would be indicative of polymer scission during chemical conjugation, we refer this phenomenon to the presence of conformers, possibly in dynamic equilibrium, that had different sizes and that was not resolved by the sizing technique.

The results in Table 1 indicate a small hydrodynamic diameter for the preconjugate P/PEG(5%)/GH(5%). Based on this and on the fact that only 5% of the polymer carboxylates was attached to DOX, we estimated that the hydrodynamic parameter for P/PEG(5%)/GH-DOX(5%) would not be larger than 7–8 nm. Such small sizes are favorable for deep diffusion into tumor tissue; however, particles of this size are washed out through the kidneys within a few hours and could be disfavoring the treatment efficacy. The low negative Zeta potential (−5.34 mV) is in the range known empirically as being non-disruptive for membranes and being optimal for cellular uptake.

**Figure 2:**
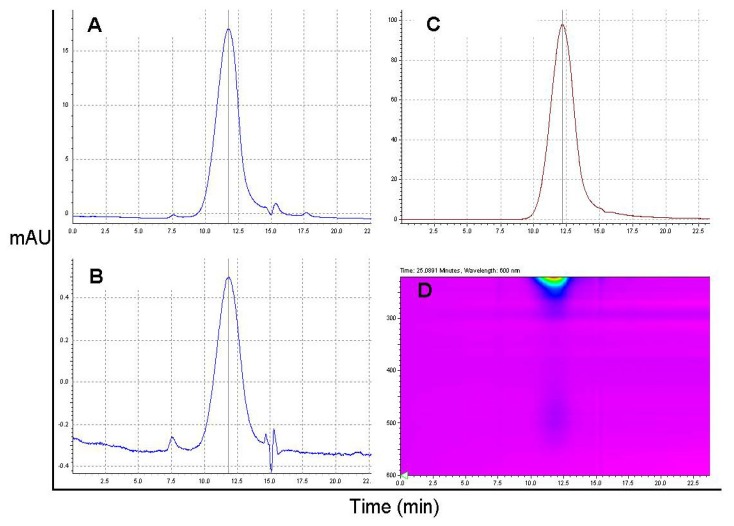
SEC-HPLC chromatograph of nanoconjugate P/PEG(5%)/GH-DOX(5%) after purification using size exclusion chromatography. Eluents were scanned at different wavelengths: (**A**) 220 nm (near the absorbance maximum for PMLA); (**B**) 480 nm (absorbance maximum for DOX); (**C**) 570 nm (DOX fluorescence, excitation at 475 nm) and (**D**) 220–600 nm wavelength range indicating degree of absorbance by a color code (red > yellow > green > blue > magenta).

Nanoconjugate P/PEG(5%)/GH-DOX(5%) was characterized by SEC-HPLC (Figure 2) and polydispersity value *P* = Mw/Mn = 1.2 (Mw, weight-averaged molecular weight; Mn, number-averaged molecular weight), which was similar to PMLA used in the synthesis, and which is at variance with the different PDI values in Table 1. This finding supports the above assumption of different conformers for the nanoconjugate in dynamic equilibrium that could have affected the high PDI-value by the light scattering technique but escaped detection by SEC-HPLC. Identical retention times at different wavelengths and absence of major other elution peaks suggested high chemical purity. The difference of 0.2 min between positions by absorbance and fluorescence (Figure 2C) corresponded with the time interval between absorbance and fluorescence detections.

### 2.3. Drug Release from the P/PEG(5%)/GH-DOX (5%) Nanoconjugate

**Figure 3:**
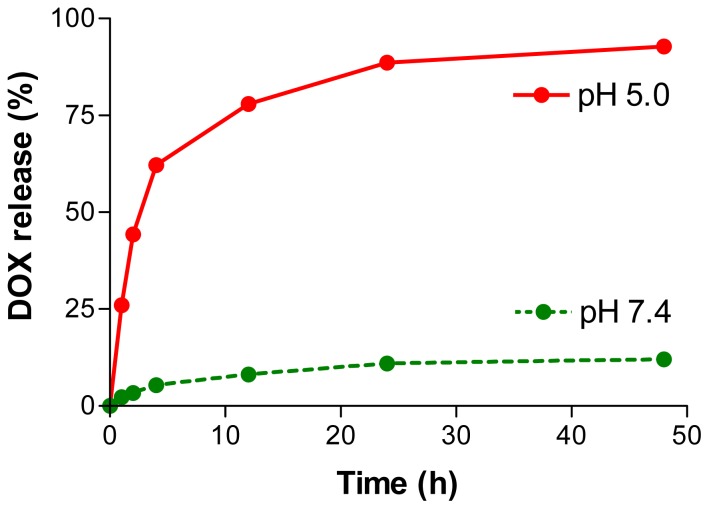
Release kinetics of DOX from nanoconjugate P/PEG(5%)/GH-DOX(5%) at pH 5.0 (red) and pH 7.4 (green), at 37 °C.

Release of DOX from nanoconjugate P/PEG(5%)/GH-DOX(5%) was measured in order to verify efficient release at pH 5.0 in mature endosome/lysosome and ideally absence of release at physiological pH 7.4 during systemic delivery. The results in Figure 3 show that nanoconjugate exhibited a steady release pattern with a tiny amount of initial burst release and a sustained release thereafter. The release at pH 5.0 was very efficient following a 50% release after 3 h and >80% after 40 h. At physiological pH 7.4, an amount of 10% of the loaded DOX was released after 40 h. This kind of release from nanoparticles is not entirely new and has been observed in many studies before [31–33]. The burst release indicated a possibility of electrostatic binding of positively charged DOX to negatively charged pendant carboxylates of PMLA platform. This amount varied with the degree of purification, but could not be suppressed completely. In contrast, the rapid release at pH 5.0 was accounted for by the acid cleavage of the hydrazone spacer that linked DOX to the nanoconjugate backbone.

### 2.4. Effect of Nanoconjugates on Cell Viability

**Figure 4:**
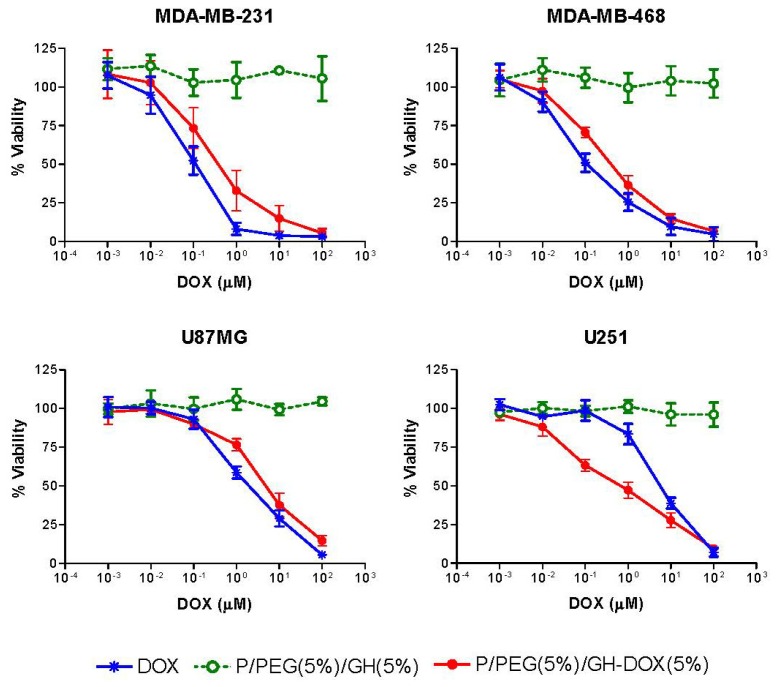
Effect of nanoconjugate P/PEG(5%)/GH-DOX(5%), P/PEG(5%)/GH(5%) and free DOX on cell viability of human breast cancer cell lines MDA-MB-231, MDA-MB-468 and human glioma cell lines U87MG, U251. The concentrations refer to DOX content.

The effects of nanoconjugate P/PEG(5%)/GH-DOX(5%) on cell viability of invasive breast carcinoma cells MDA-MB-231 and MDA-MB-468, and on human glioma cells U87MG and U251 was measured. The degree of inhibition of cell viability was compared with that of free DOX and of the carrier nanoconjugate P/PEG(5%)/GH(5%) without DOX (Figure 4). IC_50_ values are summarized in Table 2. The results indicated that the inhibition by nanoconjugate P/PEG(5%)/GH-DOX(5%) followed a dose dependant response similarly to that of free DOX, but was shifted towards 2 to 3 fold higher concentrations in the case of cell lines MDAMB-231, MDAMB-468 and U87MG, and was shifted towards lower concentrations in the case of DOX-resistant glioma cell line U251.

The empty vehicle P/PEG(5%)/GH(5%) was well tolerated by all cell lines within all used concentrations. Thus, DOX containing nanoconjugate and free DOX significantly decreased viability in all four cell lines. Interestingly, while free DOX was somewhat more effective for most cell lines, conjugated DOX P/PEG(5%)/GH-DOX(5%) was most effective on glioblastoma cell line U251 with IC_50_value of 0.8(±0.3) μM compared with IC_50_ of 5.7 (±0.8) μM for free drug. Malignant glioma is a highly aggressive form of brain cancer and has a very poor prognosis [34].

Compared with breast cancer cell lines such as MDA-MB-231 and MDA-MB-468, glioma cells were much less responsive to free DOX as was evident by IC_50_ values, 2.16 (±0.5) for U87MG and 5.7 (±0.8) U251, which were 10 to 40 times higher. Human glioma cell line U251 is known to express *P*-glycoproteins, which is associated with drug resistance [35]. The overexpression of the membrane-bound protein that affects efflux of certain drugs from cells leads to decreased intracellular concentrations of DOX [36] and hence causes low cytotoxicity. In addition, U251 has significantly higher expression of ABC transporters [37] which is another source for drug resistance. By delivering DOX via PMLA nanoplatform, the mode of drug uptake was significantly altered and bypassed the efflux mechanisms thus avoiding drug resistance. In addition to the observation of a decreased IC_50_, the concentration range of U251 toxicity spanned four orders of magnitude, while that in all the other cases covered only 2–3 orders of magnitude. This broad concentration dependence suggested that the U251 cell line consisted in a population of varying DOX-resistance. Heterogeneity in gene expression is not unusual for cancer cells and has been addressed in the issue of personalized cancer therapy [38]. In the case of U87MG cell line, the free DOX was more active than the polymer conjugated one and showed a similar trend as seen in breast cancer cell lines. This suggests that the mechanism for U251 cells to bypass resistance may not hold in this case.

DOX is primarily the drug of choice to treat breast cancer and other types of cancer but not brain tumor because of several factors, most of all including blood brain barrier. Breast cancer cell lines MDA-MB-231, MDA-MB-468 are more susceptible to free DOX as seen by the low IC_50_ values of 0.11 (±0.04) and 0.14 (±0.05) respectively. It is noticed, however, that IC_50_ values were 2–3 fold higher for the treatment with the nanopolymer version. Higher IC_50_ in comparison with free drug is not new [26] and may reflect a different route for the uptake of P/PEG(5%)/GH-DOX(5%) compared with free DOX by these cancer cells.

## 3. Experimental Section

### 3.1. Materials

DOX was purchased from AK Scientific, Inc. (Mountain View, CA, USA). PMLA (70 kDa; polydispersity 1.3; hydrodynamic diameter 5.6 nm; ζ potential −22.9 mV, pH 7.0 at 25 °C) was highly purified from culture broth of *Physarum polycephalum* as described [39]. mPEG_5000_-NH_2_ was obtained from Laysan Bio, Inc. (Arab, AL, USA). Unless otherwise indicated, all chemicals and solvents of highest purity were purchased from Sigma-Aldrich (St. Louis, MO, USA).

### 3.2. Analytical Methods Used in Chemical Synthesis

The conjugation reaction of PMLA with PEG and GBH was followed by thin layer chromatography (TLC) on precoated silica gel 60 F254 aluminium sheets (Merck, Darmstadt, Germany) and visualization of spots by UV light and/or by ninhydrin staining. Size exclusion chromatography (SEC-HPLC) was performed on an ELITE LaChrom analytical system with Diode Array Detector L 2455 (Hitachi, Pleasanton, CA, USA), using BioSep-SEC-S 3000 (300 × 7.80 mm) (Phenomenex, Torrance, CA, USA) with 50 mM sodium phosphate buffer pH 6.8 at flow rate of 0.75 mL/min. The amount of released DOX was measured at 475 nm by Reverse phase HPLC (Column: CAPCELL PAK C18, type SG 120, 5 mm, size 4.6 mm × 250 mm with acetonitrile and water (0.1% TFA) gradient with flow rate of 0.8 mL/min). The amount of DOX conjugated to PMLA was determined spectrophotometrically by absorbance measurements using coefficient (*ɛ*_480_ = 11500 M^−1^ cm^−1^).

### 3.3. Size and Zeta Potential Measurements

Hydrodynamic diameter and ζ-potential were measured at 25 °C using the Zetasizer Nano ZS90 System (Malvern Instruments, Malvern, UK). The hydrodynamic diameter of the nanoconjugates was calculated on the basis of noninvasive back-scattering (NIBS) measurements using the Stokes-Einstein equation, d(H) = kT/3πηD. d(H) is the hydrodynamic diameter, D translational diffusion coefficient, k Boltzmann’s constant, T absolute temperature, and η viscosity. The ζ-potential was calculated from the electrophoretic mobility based on the Helmholtz-Smoluchowski formula, using electrophoresis M3-PALS [40,41]. All calculations were carried out by the Zetasizer 6.0 software (version 6.0; Malvern Instruments Ltd.: Worcestershire, UK, 2008). For the nanoconjugate size measurements at 25 °C, the solutions were prepared in PBS at a concentration of 2 mg/mL and filtered through a 0.2 μm pore membrane. For measurements of ζ-potential, 1 mg/mL solutions in water containing 10 mM NaCl were prepared. The voltage applied was 150 V. Solutions were freshly prepared before analysis. Reported data are means for three independent measurements.

### 3.4. Synthesis of Nanoconjugate P/PEG(5%)/GH(5%)

*N*-Hydroxysuccinimide (NHS) (1 mmol) and DCC (1 mmol) dissolved in 2 mL of DMF were added to the solution of 116 mg of PMLA (1 mmol with regard to malyl units) dissolved in 2 mL of anhydrous acetone under vigorous stirring at RT. After stirring for 3 h to complete the activation of carboxyl groups, 0.05 mmol of mPEG_5000_-NH_2_ (in 1 mL of DMF, 5 mol% with regard to malyl units) was added followed by 0.05 mmol of triethylamine (TEA). Reaction was completed after 45 min indicated by TLC/ninhydrin tests. Next, 0.05 mmol of GBH in 1 mL DMF (5 mol% with regard to malyl units) was added at RT followed by 0.05 mmol of TEA. The reaction was completed within 2 h according to TLC. Addition of 5–6 mL of 100 mM sodium phosphate buffer containing 150 mM NaCl (pH 5.5) was followed by 1 h stirring at RT to remove Boc. After centrifugation at 1500*g* for 10 min, the clear supernatant containing glycine hydrazide (GH) was passed over a Sephadex column (PD-10, GE Healthcare, Piscataway, NJ, USA) pre-equilibrated with deionized (DI) water. Fractions containing the product P/PEG(5%)GH(5%) were combined and lyophilized to obtain a white solid.

### 3.5. Synthesis of Nanoconjugate P/PEG(5%)/GH-DOX (5%)

To a solution of P/PEG(5%)/GH(5%), 10 mg in 2 mL DMF, was added a solution of excess DOX, 2 mg in 1 mL of DMF, along with activated molecular sieves (4 Å, 10–18 mesh). The reaction mixture was stirred in the dark for 16 h, before phosphate-buffered saline (PBS, pH 7.4) 6 mL was added. The clear supernatant was passed over a Sephadex column (PD-10, GE Healthcare, Piscataway, NJ, USA) pre-equilibrated with PBS pH 7.4. The P/PEG(5%)/GH-DOX(5%) fractions were collected and immediately frozen using liquid nitrogen. After lyophilization, conjugate P/PEG(5%)/GH-DOX(5%) was obtained as a pinkish red solid.

### 3.6. Release of DOX from the Nanoconjugate

The amount of DOX released from the polymer conjugate was measured by HPLC Hitachi system of samples directly taken during incubation of polymers in sodium acetate buffers (0.1 m sodium acetate with 0.05 M NaCl, pH 5.0 or 7.4) at 37 °C. The peak intensity at 475 nm was directly proportional to the amount of DOX released from the polymer conjugate. The calibration curve was carried out using known concentration of DOX. All drug-release data are expressed as the amounts of free DOX relative to the total DOX content in the conjugate.

### 3.7. Cell Lines and Culture Conditions

Invasive human breast carcinoma cell lines MDA-MB-231 and MDA-MB-468 and human primary glioma cell lines U87MG and U251 were obtained from American Type Culture Collection (ATCC, Manassas, VA, USA). U87MG was cultured in minimum essential media (MEM) supplemented with 10% fetal bovine serum, 1% MEM NEAA, 1 mM sodium pyruvate and 2 mM l-glutamine. MDA-MB-231, MDA-MB-468 and U251 were cultured in Dulbecco’s Modified Eagle Medium (DMEM) supplemented with 4.5 g/L d-glucose, l-glutamine and 10% fetal bovine serum. Cells were seeded at 5000 per well (0.1 mL) in 96-well flat-bottomed plates and incubated overnight at 37 °C in humid atmosphere with 5% CO_2_. After exposure to nanoconjugates for 48 h, medium was replaced with fresh media, and cell viability was measured using Cell Titer 96 Aqueous One Solution Cell Proliferation Assay kit (Cat. No. PR-G3580; Promega, Madison, WI, USA). The yellow reagent (3-(4,5-dimethylthiazol-2-yl)-5-(3-carboxymethoxyphenyl)-2-(4-sulfophenyl)-2*H*-tetrazolium, inner salt) (MTS) is bioreduced by viable cells yielding formazan that is read by a spectrophotometer. The absorbance reading at 490 nm from the 96-well plates is directly proportional to the number of living cells [42]. The viability of the untreated cells was taken as 100%. The results shown are means ± average deviation of three independent measurements. Data were analyzed by statistical software GraphPad Prism 3.0 (version 3.0; GraphPad Software Inc.: San Diego, CA, USA, 1999).

## 4. Conclusions

For the purpose of delivering DOX to human cancer cells, DOX was successfully conjugated to a PMLA platform via an acid labile hydrazone bond. The majority of DOX (>80%) was released from the PMLA-platform under acidic pH prevalent in late endosome and lysosomes. A slow release of DOX was observed at physiological pH 7.4. Since most of DOX (~90%) still remained bound to the platform after 40 h, drug delivery *in vivo* was considered efficient and the level of cytotocitity low due to clearance of the nanoconjugate from the blood during this time. DOX containing nanoconjugate showed significant reduction in tumor cell viability of both human glioma and human breast cancer cell lines, whereas the PEG containing platform in the absence of DOX was inactive. The results indicated that DOX containing nanoconjugates of PMLA were active comparably to free DOX in arresting cancer cell viability and could be used as safe delivery vehicles to treat brain and breast cancer. Conjugates P/PEG(5%)/GH-DOX(5%) containing targeting monoclonal antibodies will be designed for *in vivo* targeted delivery of DOX as new versions of the Polycefin family to treat brain tumors.

## Figures and Tables

**Scheme I:**
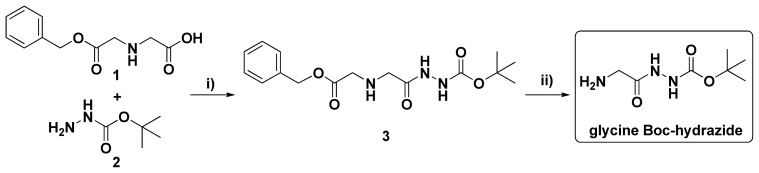
Synthesis of glycine Boc-hydrazide. Reagents and conditions: (**i**) *N*,*N*′-Dicyclohexylcarbodiimide (DCC), Ethyl acetate, 0 °C 2 h, RT 4 h, yield 78%; (**ii**) H_2_/Pd-C, MeOH, RT 16 h, yield 80%.

**Scheme II:**
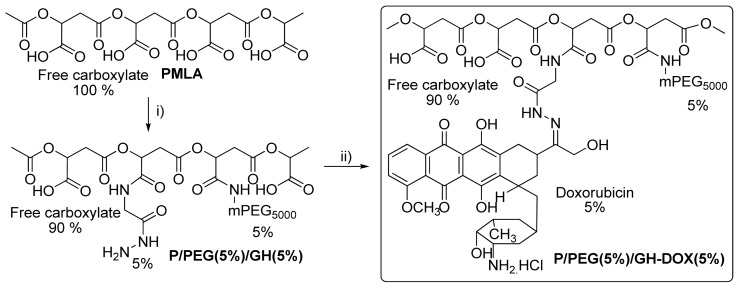
Synthesis of P/PEG(5%)/GH-DOX(5%). Reagents and conditions: (**i**) Mixture of NHS and DCC, Acetone, DMF, RT 3 h, followed by addition of mPEG_5000_-NH_2_, RT 45 min, and by glycine hydrazide, 2 h yield 67%; (**ii**) DOX.HCl, molecular sieves 4 Å, 10–18 mesh, DMF, RT 16 h, yield 72%.

**Table 1:** Physicochemical properties of nanoconjugates.

Nanoconjugates	Size in nm	Zeta Potential in mV	Polydispersity Index (PDI)	Molecular Weight in kDa
PMLA	5.6 (±0.1)	−22.9	0.1 (±0.01)	70
P/PEG(5%)/GH(5%)	6.2 (±0.5)	−11.45	0.64 (±0.03)	223
P/PEG(5%)/GH-DOX(5%)	nd	−5.34	nd	239

nd: not determined (interference of DOX fluorescence with scattered light detection).

**Table 2:** Effect of free and conjugated DOX on cell viability.

Cell Lines	DOX IC_50_ (μM)	P/PEG(5%)/GH-DOX(5%) IC_50_ (μM)
MDA-MB-231	0.11 (±0.04)	0.52 (±0.2)
MDA-MB-468	0.14 (±0.05)	0.51(±0.1)
U87MG	2.16 (±0.5)	5.73 (±2.1)
U251	5.7 (±0.8)	0.8 (±0.3)
